# “Thermal Stabilization Effect” of Al_2_O_3_ nano-dopants improves the high-temperature dielectric performance of polyimide

**DOI:** 10.1038/srep16986

**Published:** 2015-11-24

**Authors:** Yang Yang, Jinliang He, Guangning Wu, Jun Hu

**Affiliations:** 1 State Key Laboratory of Power System, Department of Electrical Engineering, Tsinghua University, Beijing 100084, China; 2 School of Electrical Engineering, Southwest Jiaotong University, Chengdu 610031, China

## Abstract

Insulation performance of the dielectrics under extreme conditions always attracts widespread attention in electrical and electronic field. How to improve the high-temperature dielectric properties of insulation materials is one of the key issues in insulation system design of electrical devices. This paper studies the temperature-dependent corona resistance of polyimide (PI)/Al_2_O_3_ nanocomposite films under high-frequency square-wave pulse conditions. Extended corona resistant lifetime under high-temperature conditions is experimentally observed in the 2 wt% nanocomposite samples. The “thermal stabilization effect” is proposed to explain this phenomenon which attributes to a new kind of trap band caused by nanoparticles. This effect brings about superior space charge characteristics and corona resistance under high temperature with certain nano-doping concentration. The proposed theory is experimentally demonstrated by space charge analysis and thermally stimulated current (TSC) tests. This discovered effect is of profound significance on improving high-temperature dielectric properties of nanocomposites towards various applications.

Polymer/nano-filler composites have attracted a great deal of interest for scientific researches and industrial applications in many fields including aerospace, biomedicine, structural materials, electronic and electrical (or power) engineering. Nanocomposites filled with nano-dopants can significantly improve the performance of the polymers and sometimes bring novel mechanical, thermal and electrical properties to overcome the low operating temperature of polymer matrixes. The application of nanocomposites in electrical insulation field brings about the concept of nanodielectrics which was first theoretically raised by Lewis (1994)^1^. Recent researches demonstrated that nano-fillers (usually oxide nanoparticles) impart decreased permittivity^2^,^3^, enhanced breakdown strength^4^,^5^, corona resistance^6^ and thermal-mechanical performance^7^ to the original polymer dielectrics especially under high-temperature conditions. Nowadays, using nano-fillers to tailor the properties of nanodielectrics has become a popular and effective strategy to develop dielectrics and many other engineering materials with specific high performance. However, the mechanism of nano-modification is still an open issue in consideration of the complex nanostructure and characteristics of phase interface^8^,^9^. The novel properties of nanocomposites are thought to be dominated by the interface^10^,^11^. Many researchers have tried a variety of methods to probe and characterize these small (few tens of nm) but significant regions^12^,^13^. According to the researches mentioned above, various models focused on the structure and characteristics of phase interface have been carried out including DLVO (Derjaguin and Landau, Verwey and Overbeek) model^14^, multi-core model^15^ and water shell model^16^. Nevertheless, these physical or chemical descriptions are not clear enough to explain all the experimental results and the temperature condition has not been taken into account. The design of nanodielectrics is still largely based on experience. Phase interface models of the nanocomposites should be further improved to provide the reliable theoretical basis for engineering practice.

In insulation system design of electrical devices, how to improve the dielectric performance of insulation materials under high-temperature conditions is one of the key issues^8^,^17^,^18^. Polyimide (PI) has been gaining increasing attention in various insulating applications (e.g., insulation of traction motor windings, microelectronics, flexible electronics, etc.) due to its outstanding dielectric performance as well as excellent thermal and mechanical characteristics^19^,^20^,^21^,^22^. Nano doping of ceramic oxides (such as Al_2_O_3_, TiO_2_, SiO_2_, etc.) can greatly enhance the dielectric properties of PI composite and maintain high thermal endurance^19^,^20^,^21^,^22^,^23^. Some kinds of ceramic nano-dopants in PI matrix provide better dielectric properties at higher temperature, which have been used to improve the insulation of traction motor windings. Recently, Zha *et al.* found that nano-TiO_2_ particles could improve the dielectric strength, lifetime, and space charge characteristics based on the dielectric properties tests of PI/TiO_2_ nanocomposites^19^,^20^. Zhou *et al.* attributed the enhanced corona resistance of PI nanocomposites to the high charge transport capacity of nanostructure based on the corona-aging and breakdown tests under high frequency square wave pulses^21^,^24^. However, most of the researches focus on the comparison between pure PI and nanocomposites at the same temperature. Few in-depth explanations of this effect, that the nanoparticles improve the insulating properties of polyimide at high temperature, have been proposed up till now, and the influential mechanism is still uncertain.

In this research, an extension of corona resistant lifetime is observed under high-temperature conditions with regard to the PI samples doped with a certain concentration (2 wt%) of nano-Al_2_O_3_. In order to explain this unexpected phenomenon, the “thermal stabilization effect” is proposed based on the space charge analysis by pulsed electro acoustic (PEA) method. The thermally stimulated current (TSC) tests further demonstrate the synergistic effect of nano-doping concentration and temperature on nanoscale electronic transport. Based on the results of PEA and TSC tests, the underlying mechanism of this effect along with its nano-doping-concentration and temperature dependence is interpreted in reference to the “multi-core” model with an innovative temperature-dependent nanostructure of phase interface introduced. This improved model reveals a candidate theoretical foundation for refined nanodielectrics design with the optimal nano-doping concentration under high-temperature conditions of various electrical insulation applications. The proposed “thermal stabilization effect” is essentially bound up with the electronic transport in nanoscale system of polymer nanocomposites which not only affect the corona resistance but dominate the dielectric response, conductivity, electrical breakdown and dielectric loss of polymer nanocomposites^1^,^9^. Thus this effect is a general phenomenon in dielectrics and electrical insulation. In addition, further investigation about this effect is of great significance in characterizing and modeling the phase interface regions of polymer nanocomposites.

## Results and Discussion

### Characterization of chemical structures and micro-morphology

PI/Al_2_O_3_ nanocomposite films with 0 wt%, 2 wt% and 5 wt% nano-Al_2_O_3_ (30 nm) are prepared by *in-situ* polymerization. Nanoparticles are surface modified with silane coupling agent KH550 and dispersed by ultrasound (Method section). Pyromellitic dianhydride (PMDA) and 4,4′-Oxdianline (ODA) are chosen as the monomers to prepare Kapton (Method section), a kind of PI first developed by DuPont. Fourier transform infrared spectroscopy (FT-IR, Thermo Nicolet 5700, USA) tests are carried out to analyze the chemical structure of the PI/Al_2_O_3_ nanocomposite films (Supplementary Note 1, Fig. S1). The FT-IR spectrum curves of the nanocomposite films demonstrate the completed polymerization and imidization reaction of the samples. Micro morphologies of the films are observed by field emission scanning electron microscope (FE-SEM, ZEISS Sigma, Germany) (Supplementary Note 2, Fig. S2). The surface SEM image demonstrates the good surface smoothness of the synthesized film which is crucial for corona-aging tests. From the eroded cross-section SEM image (see Method section), it is observed that the nanoparticles are below 100 nm in size and show homogeneous distribution.

### Corona-aging lifetime

In the corona-aging tests, the rode-plate electrode structure, the corona-aging area and the experimental equipment are shown in Supplementary Note 3, Fig. S3 (a), (b), and Fig. S4, respectively. The PI/Al_2_O_3_ nanocomposite samples and electrodes are cleaned with anhydrous alcohol, then desiccated in the vacuum oven at 120 °C for 2 hours before corona-aging tests to avoid the influence of water absorption on the dielectric behavior of PI^25^. The running temperature of inverter-fed motor polyimide insulation reaches 140 °C^26^ or higher during the practical operation and the polyimide films in many power electronic devices including insulated gate bipolar translator (IGBT) have to sustain the operating temperature of 150 °C^27^. Thus the experiments are carried out on 0 wt%, 2 wt% and 5 wt% PI/Al_2_O_3_ nanocomposites films at 80 °C, 120 °C and 160 °C, respectively. Previous research indicated that the space charge accumulation threshold fields of common polyimide film-100HN and corona-resistant polyimide film-100CR (both are DuPont products, the former is pure PI film and the latter is PI/Al_2_O_3_ nanocomposites film) are 31.5 kV/mm and 35 kV/mm^21^, respectively. In order to study the influence of space charge accumulation on corona resistance, the tested electrical fields are above 30 kV/mm and slightly higher than the referenced threshold fields mentioned above. The applied square-wave voltage is adjusted according to the thickness of the samples to set the electric fields as 30 kV/mm, 33 kV/mm, 36 kV/mm, 39 kV/mm and 42 kV/mm (peak-to-peak value). The frequency of the HV square wave pulse is fixed at 10 kHz during all the tests. All the parallel experiments are repeated four times to obtain the error bar data.

**Figure 1:**
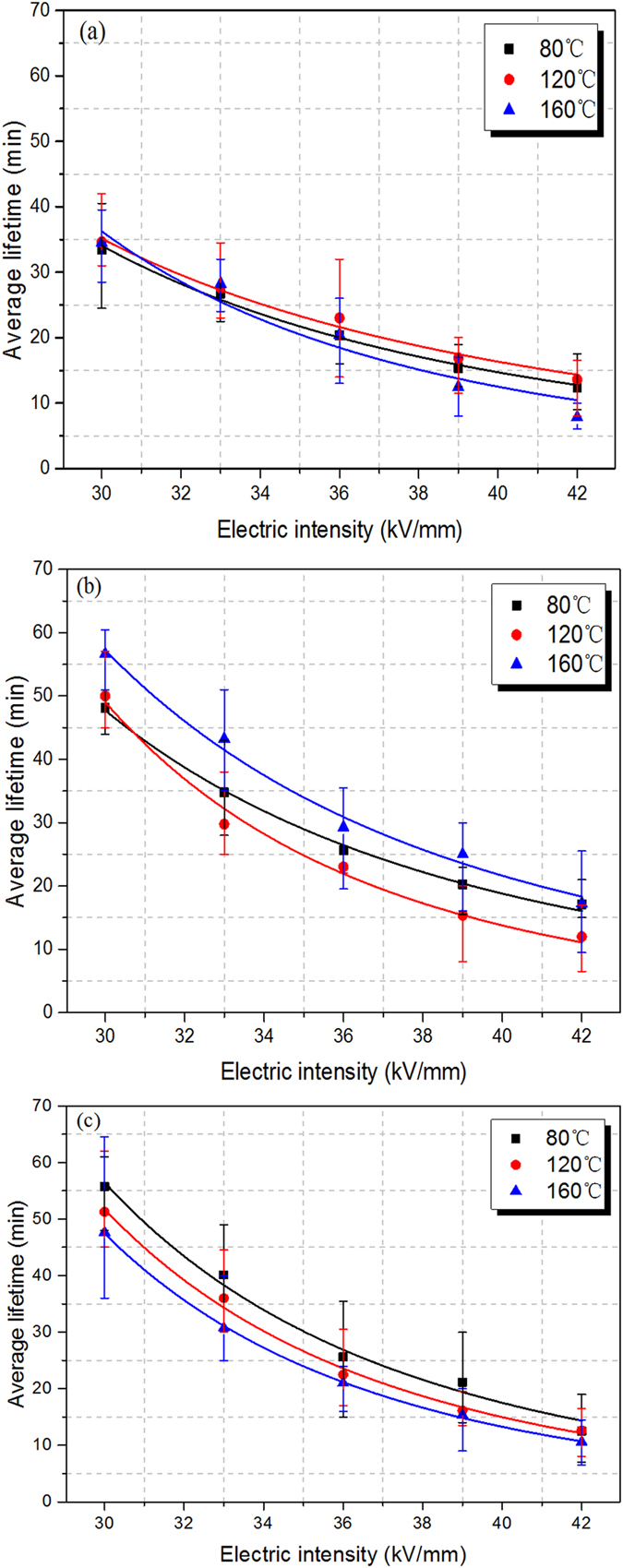
The relationship between the corona resistant lifetime and the average electric field intensity from the corona-aging tests of different kinds of nanocomposite films at different temperatures. The curves are fitted by the least square method with power type and the error bars show the maximum and minimum value among the four repeated tests. (**a**) Pure PI (0 wt%) films. (**b**) 2 wt% PI/Al_2_O_3_ nanocomposite films. (**c**) 5 wt% PI/Al_2_O_3_ nanocomposite films.

From the corona-aging tests, the relationship between the lifetime and the average electric field intensity is shown in Fig. 1. The corona resistant lifetime *L* is related to applied voltage^28^,^29^, and the relationship can be further derived as an inverse power model of




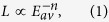




where *E*_av_ is the average electric field intensity, and *n* is a constant related to material property and test conditions (Supplementary Note 4).

The lifetime of PI/Al_2_O_3_ nanocomposite films is much higher than the pure PI films especially under low field (near 30 kV/mm) and Al_2_O_3_ nanoparticles effectively improve the corona resistance of nanocomposite films. From 80 °C to 120 °C, a little improvement of the lifetime is obtained through all the electrical field strengths regarding the pure PI samples. This might be due to the mobility enhancement of charge carriers under high temperature which restrains the accumulation of surface charge and remits the local electric field enhancement. At 160 °C, the lifetime shows higher dependence on the applied electric field strength and reduces to the values lower than the 80 °C ones. This is caused by the thermal aging of the polymer dielectrics. Because the *L*-*E*_av_ curves indicate the synthetic effect of temperature on the charge mobility and the dielectric properties of pure PI films, the dependence of lifetime on the temperature condition is not very obvious. The lifetime of the nanocomposite samples (2 wt% and 5 wt%) shows stronger dependence on temperature in comparison with the pure PI samples. At 80 °C and 120 °C conditions, 5 wt% nanocomposite films show better corona resistant performance than the 2 wt% films. However, the 2 wt% and 5 wt% films show remarkably different dependence of corona resistance on temperature. As the temperature increases from 80 °C to 160 °C, the insulation lifetime of the 2 wt% samples presents non-monotonic variation trends, while the insulation lifetime of the 5 wt% samples keeps decreasing. At 160 °C, the lifetime of 2 wt% samples increases to the values higher than the 5 wt% ones under all the tested electrical field strengthes. Al_2_O_3_ nano-dopants not only greatly improve the corona resistance of nanocomposite films but give rise to some new temperature-dependent dielectric properties. Temperature increment brings the 2 wt% films unexpected enhancement of corona resistance, which is described as the “thermal stabilization effect” here. In fact, similar phenomenon and effects, that the nanocomposites materials show superior dielectric properties under high-temperature conditions, are widespread in recent researches regarding dielectric spectrum, dielectric loss^30^,^31^, electrical breakdown and high-field capacitive energy storage properties^18^. All of these properties above are dominated by the electronic transport in nanoscale system of polymer nanocomposites which will be discussed in details below.

### Space charge distribution

In order to illustrate the mechanism of this phenomenon, the space charge distribution is measured by pulsed electro acoustic (PEA) method. The long term corona-aged samples are treated under 1 kHz square wave pulse for 4 hours with the peak-to-peak electric field density of 32 kV/mm so that the corona-aging process is much mitigated and the degree of corona-aging is easy to adjust. The space charge distribution is measured under 20 kV/mm to analyze the carrier trapping of shallow traps. Details of the PEA measurement system and the calculation of space charge density refer to ref. 32.

**Figure 2:**
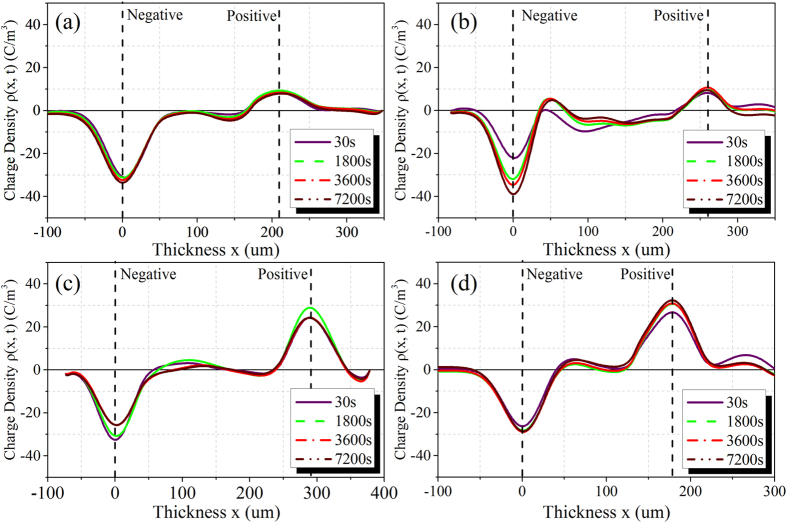
Space charge distribution (measured by PEA method) of long term corona-aged pure PI and PI/Al_2_O_3_ nanocomposite films under polarization electric field of 20 kV/mm for 2 hours. The samples are treated under high-frequency high-voltage pulse at different temperatures: (**a**) Pure PI films corona-aged at 80 °C. (**b**) Pure PI films corona-aged at 160 °C. (**c**) 2 wt% nanocomposite films corona-aged at 80 °C. (**d**) 2 wt% nanocomposite films corona-aged at 160 °C.

As is shown in Fig. 2, few hetero-charge indicates that there are nearly no ionic carriers, which is generated by ionization of chemical impurities within the matrix, and the space charge is formed due to the injected homo-charge (electron/hole) carriers at the electrodes. Figure 2(a,b) indicate that the electronic injection and accumulation (near the negative electrode) of pure PI films corona-aged at 160 °C are much severer than the samples corona-aged at 80 °C. In regard to the films corona-aged at 80 °C, as shown in Fig. 2(a,c), more electrons are injected into 2 wt% nanocomposite samples than pure PI ones. It can be assumed that nano-dopants introduce a new trap band with lower energy level and cause electronic trapping under low field. From Fig. 2(c,d), as the corona-aging temperature varies from 80 °C to 160 °C, the electronic injection and accumulation of PI/Al_2_O_3_ nanocomposite films are mitigated. The comparison between pure PI films and 2 wt% nanocomposite films corona-aged at 160 °C indicates the obvious mitigation of space charge injection of PI/Al_2_O_3_ nanocomposite films at high temperature except for the slight charge injection caused by the newly introduced shallow traps as mentioned above. Nano-dopants restrain the injection and accumulation of electrons then alleviate the local electric field which mitigates partial discharges and improves the corona resistance^21^,^33^,^34^ especially under high temperature conditions.

### Energy level of carrier traps

**Figure 3:**
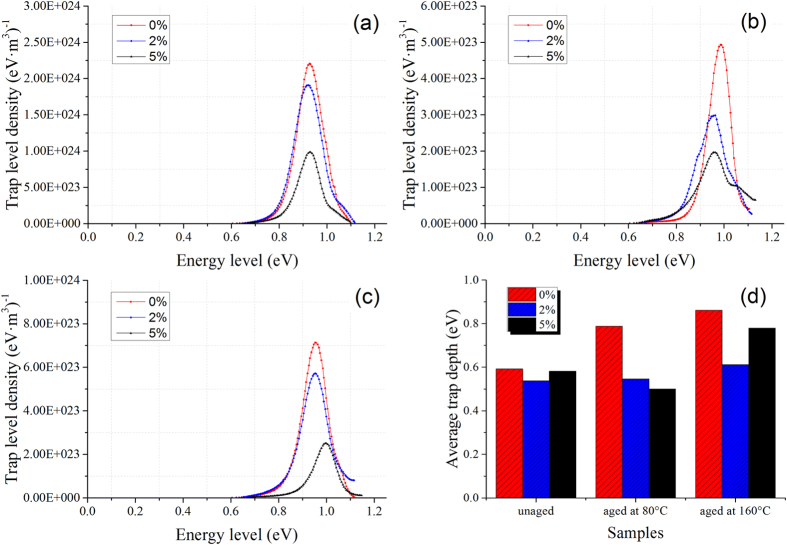
The trap level density distribution curves of PI/Al_2_O_3_ nanocomposite films with different nano-doping concentrations and temperature conditions of long term corona-aging. (**a**) Unaged samples. (**b**) Samples corona-aged at 80 °C. (**c**) Samples corona-aged at 160 °C. (**d**) The trap depth of the samples calculated the half-width method.

Thermally stimulated current (TSC) of the long term corona-aged films are measured to further prove the change of energy levels of carrier traps (details are shown in Method section). The trap level density distribution of the samples can be obtained from the thermally stimulated depolarization current (TSDC) density by numerical calculation method^35^ (Supplementary Note 5) and the trap level density distribution curves are shown in Fig. 3. The numerical differences of the trap level densities between the tested samples are caused by the different thicknesses of the films which are unavoidable during the preparation process. However, the quantity variance of the traps would not make too much influence on the following discussion and it is the change of the density distribution that make difference. In order to illustrate the energy level changes of the samples corona-aged under different conditions, the average trap depths of the samples can be estimated through the half-width method^36^ because the origin of depolarization current is the injection of electrons/holes (homo-charge, as demonstrated by PEA tests):




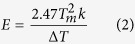




where *T*_*m*_ is the temperature corresponding to the peak value of TSC curves, Δ*T* is the width of the current curve at half peak and *k* is the Boltzmann constant. The average trap depths calculated by the half-width method are shown in Fig. 3(d) and Table 1.

Figure 3 illustrates that the trap levels of the samples distribute between 0.6 eV and 1.2 eV and show a peak similar to the TSDC curves (Supplementary Note 5). The peak levels of the unaged samples are 0.928 eV, 0.919 eV and 0.924 eV for 0 wt%, 2 wt% and 5 wt% PI/Al_2_O_3_ nanocomposite films, respectively. The average trap depths calculated by half-width method also show the same variation trend. The nano-dopants drag the peak of trap level density curves to the lower energy levels or the PI/Al_2_O_3_ nanocomposite films introduce a new trap band with lower energy level comparing with the pure PI films. Nevertheless, the changes are not obvious because the TSDC is almost contributed by the detrapping of the charge carriers within a finite depth (typically only 5 μm)^35^ of the films adjacent to the electrodes and the nanofiller concentration near the surface of the film is always lower than the inside for the prepared nanocomposite films. Regarding to the long term corona-aged samples, the trap level density curves and the average trap depths show significant differences because the aforementioned thin surface layer of the films are corroded by corona discharge and the inner nanocomposites can be tested. Higher corona-aging temperature results in deeper charge carrier traps in pure PI films corresponding to chemical or physical defects formed by corona erosion. The trap level of the corona-aged samples reasonably indicate the degradation degree which reflects the corona resistance of the dielectrics. After corona-aged at 80 °C (Fig. 3(b)), the trap level density of the 2 wt% film shows a small overlapped peak with low energy level (<0.9 eV) and indicates the shallow traps introduced by the nanoparticles. There is also a small high energy level peak in the 5 wt% sample’s curve, this might be due to the small amount of defects formed by the aggregation of high concentration nanoparticles. As is demonstrated by the results of corona-aging lifetime tests, this slight aggregation of nanoparticles does not degrade the dielectric performance of the films and the average trap depth decreases. Nanocomposite films show lower trap energy level and lower degradation degree comparing with pure PI films, and the average trap depth of the 5 wt% sample is lower than the 2 wt% sample. The high nano-dopants concentration films contain more shallow traps which agrees well with the results and the assumption in “space charge distribution” section. As the temperature conditions of corona-aging rise to 160 °C, the trap level density peak of the 5 wt% sample moves to the higher level and the average trap depth increases a lot which indicates severe degradation of the film. The trap energy level of corona-aged nanocomposite films with 5 wt% nano-Al_2_O_3_ becomes lower (aged at 80 °C) and then rises higher (aged at 160 °C). The peak value and average trap depth of the 2 wt% sample maintain at a low level which indicates subdued degree of corona-aging. The 2 wt% nanocomposite film exerts better corona-resistant performance than the 5 wt% one at high temperature which is in correspondence with the results of corona-aging lifetime tests. Higher concentration (5 wt%) of nanofiller contributes to better dielectric properties at low temperature (80 °C) while showing serious degradation and sacrificing the “thermal stabilization effect” at high temperature (160 °C).

The aforementioned phenomenon appears in a certain range of nano-doping concentration at a certain range of temperature which indicates that the corona-resistance and space charge characteristics of PI/Al_2_O_3_ nanocomposite films not only depend on the nano-doping-concentration but show some temperature-dependence. The improvement of dielectric performance and space charge characteristics introduced by a certain concentration of nano-dopants has been studied by many researchers with some discrepancy among their results and conclusions^9^,^33^. Up till now, few of them take the temperature condition into account and this may be one of the main influence factors. To further give a comprehensive description about the various behaviors of polymer-nanofiller system under different nano-doping-concentration and temperature conditions, the “thermal stabilization effect” of the nano-dopants is interpreted below.

### Mechanisms of the “thermal stabilization effect”

Based on the theory of interphase and “interaction zones”, Tanaka proposed the “multi-core” model to explain the interaction mechanism between nanofiller and polymer matrix^8^,^9^. The model describes the phase interface as a three-layer shell structure overlapped with an electric double layer, and it attributes the reduction of space charge accumulation to the new traps in the phase interface, especially in the loose layer (or the third layer)^9^. Compared with the original traps in the polymer matrix, these new traps are shallower (with lower energy level) because these regions have high free volume and low density. The shallow traps will not hold the electronic carriers for a long time^37^. On the contrary, they would help the carriers to transport then lead to high mobility of the electronic carriers and mitigate space charge accumulation^9^.

**Figure 4:**
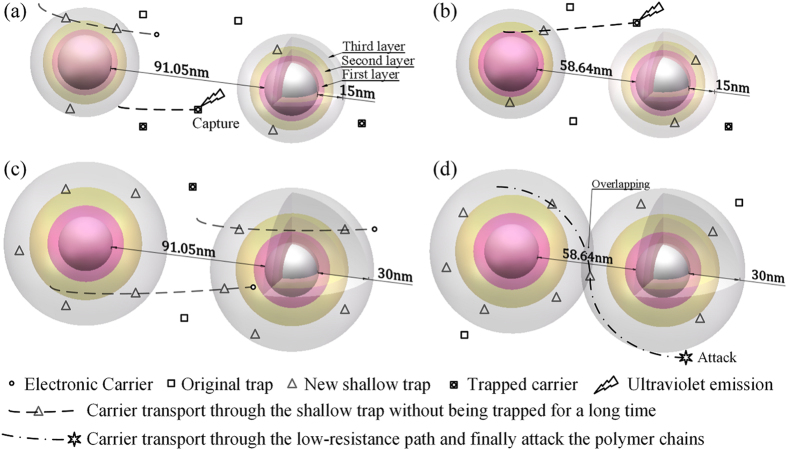
Nano-dopants concentration and temperature determine the nanostructures of phase interface based on “multi-core” model: (**a**) 2 wt% nanocomposites show small shallow-trap regions at 80 °C.(**b**) 5 wt% nanocomposites show large shallow-trap regions and low carrier mobility at 80 °C. (**c**) 2 wt% nanocomposites show large shallow-trap regions and high carrier mobility at 160 °C which help carriers transport. (**d**) 5 wt% nanocomposites show large shallow-trap regions and overlapping low-resistance paths at 160 °C which causes high energy carrier to attack polymer chains.

However, the nanostructures introduced by different nano-doping concentrations may be entirely different. Because of the low nano-dopants concentration (not more than 5 wt%), the nanoparticles in polymer matrix are incompact and the spatial positions of the nanoparticles can be treated as a simple cubic stacking system which gives the lowest space utilization. As is shown in Fig. 4, according to the density of PI (Kapton, 1.42 g/cm^3^), α-Al_2_O_3_ (true density, 3.6 g/cm^3^) and particle diameter (30 nm), the inter-particle distance (surface to surface) can be calculated as 91.05 nm and 58.64 nm for 2 wt% and 5 wt% in simple cubic stacking, respectively. Here the density decrease of low density region is ignored. The temperature-dependent chain mobility would change the structure of phase interface. The total thickness of three-layer shells is 10 to 30 nm and the third layer shell of “multi-core” is characterized by many factors with strong temperature dependence (e.g., chain mobility)^8^.

As is illustrated in Fig. 4, nanostructures introduced by different nano-dopants concentration and temperature conditions would result in various electronic carrier transport behaviors. It is assumed that, as the temperature increases from 80 °C to 160 °C, the thicknesses of three-layer shells will be extended. In the case of low nano-doping concentration and low temperature represented in Fig. 4(a), the volume fraction of phase interface is small and electrons are more likely to be captured by the original (deep) traps accompanied with ultraviolet emission^38^ which would lead to further degradation of the matrix. In Fig. 4(b,c), large volume fraction of phase interface is introduced by small inter-particle distance and large layer shell thickness, respectively. There are more electrons trapped in deep traps in (b) compared with (c) because electrons show higher mobility at higher temperature. If the inter-particle distance is not large enough, regarding to the 5 wt% samples, the shells would overlap with each other at higher temperature, as presented in Fig. 4(d). Then a loose-layer path (the dot dash line) is formed for the electrons to transport. Considering the high-free-volume low-density characteristics, these loose-layer paths provide long mean free path for electrons^39^. Electrons are likely to choose these low-resistance paths and get more energy from the electrical field. If the electronic carriers are accelerated to an enough high speed, these high energy electrons would break the molecular chains and result in structural defects of high density in the polymer matrix which would act as deep traps and accumulate space charges^40^,^41^. This can explain the formation of extra deep traps in the 5 wt% samples corona-aged at high temperature (160 °C) as shown in TSC results. The trap density distribution and the trap depths calculated by the aforementioned half-width method reasonably confirm the above interpretation.

In consequence, the increment of temperature may not only contribute to the formation of shallow-trap region but helps build the low-resistance paths for electrons as well in the case of short inter-particle distance. On the basis of the mechanism proposed above, the electronic carriers are more likely to be accelerated in the loose-layer than in the matrix. If the inter-particle distance is larger than twice of the layer shell thickness to avoid overlapping of the loose-layers, electrons would be transported through the low-density regions (the phase interface) and the high-density regions (the matrix) alternately. Then the kinetic energy of the electrons is limited within a range determined by the ratio between low-density path and high-density path or the volume fraction of the phase interface regions on a certain condition. An appropriate electron migration rate (kinetic energy) helps restrain the trapping of electronic carriers without damaging the matrix. The inter-particle distance and the interface thickness are determined by nano-dopants concentration and temperature, respectively. It is easy to consider the optimal nano-dopants concentration which may bring the most satisfactory space charge characteristics at a certain temperature. The aforementioned mechanisms of the “thermal stabilization effect” provide the probable descriptions of electronic transport in nanoscale system of polymer nanocomposites. The estimated nanostructures of the phase interface based on the “multi-core” model reasonably conform the results of space charge distribution (PEA) tests and TSC tests.

## Conclusions

The “thermal stabilization effect” based on the space charge analysis and thermally stimulated current (TSC) tests has been proposed to explain the extension of corona-aging lifetime of 2 wt% PI/Al_2_O_3_ nanocomposite films under high-temperature conditions. The results of TSC tests agree reasonably well with the nano-doping-concentration dependence and temperature-dependent mechanism of this effect which is first interpreted here in reference to the “multi-core” model. In summary, certain-concentration nano-doping of Al_2_O_3_ not only improves the high frequency high voltage (HV) square wave corona resistance of PI nanocomposites but brings the “thermal stabilization effect” of nanoparticles as well and endows PI/Al_2_O_3_ nanocomposites with better insulating performance at higher temperatures.

The interpretation of the “thermal stabilization effect” is based on the discussion about the electronic transport in nanoscale system which plays the dominate role in the electrical properties of polymer nanocomposites considering the large surface area of nanoparticles and the novel characteristics of the interface regions^1^. Similar analysis can be performed to explain the non-monotonic temperature and nano-dopants concentration dependence of dielectric spectrum, dielectric loss and conductivity for a variety of polymer nanocomposites. The proposed temperature dependence of the interface nanostructure along with its influence on the behavior of electronic carriers provides a candidate theoretical foundation for a more accurate model to characterize the phase interface region which is still an open issue in the field of polymer nanocomposites. In practical application, the extreme operating condition is usually accompanied by high temperatures and polymer-based dielectrics are usually limited for their relatively low working temperatures^18^. This improved model contributes to design high-performance polymer nanodielectrics especially for the application of high-temperature conditions. For some direct examples, regarding the PI/Al_2_O_3_ nanocomposites, “thermal stabilization effect” would minimize the insulation space for more winding turns and raise the operating temperature then improve the power density of electrical machine for electric vehicles etc. The potential optimal nano-dopants concentration aiming at the high running temperature would significantly improve the insulating performance and heat resistance of PI films applied in power electronic modules and many other microelectronic devices^42^,^43^. Further study of this effect and its generalized investigation in the field of polymer nanocomposites would be of great significance for electrical insulation and many other applications.

## Methods

### Surface modification of Al_2_O_3_ NPs

To modify the nanoparticles, about 0.1 g (1% the quality of nano-Al_2_O_3_) silane coupling agent KH550 (Sinopharm Chimical Reagent Co.,Ltd, China) is added to the alcohol (95 ml) water (5 ml) solution dispersed with about 10 g nano-Al_2_O_3_ (α phase, 30 nm, Aladdin Industrial Inc. China). Then ultrasonic treatment for 30 min at room temperature. After that, keep the solution at 70 °C in water bath and magnetically stir for 6 h to obtain the modified nanoparticles suspension. The suspension is then centrifuged (6000 rpm, 6 min) and the supernatant liquid is removed. Finally the modified nanoparticles are dried at 50 °C in the vacuum oven for 12 h and grinded into nanoparticles powder.

### Synthesis of PI/Al_2_O_3_ nanocomposite films

Stoichiometric 4,4′-Oxdianline (ODA, Sinopharm Chimical Reagent Co.,Ltd, China) and the surface modified Al_2_O_3_ nanoparticles are added into a certain amount of N,N-Dimethylacetamide (DMAc, Sinopharm Chimical Reagent Co.,Ltd, China). After ultrasonic treatment for 60 min at room temperature, pyromellitic dianhydride (PMDA, Sinopharm Chimical Reagent Co.,Ltd, China) is added into the solution and the mole ratio n (PMDA)/n (ODA) = 1.01. Then magnetically stir the solution for 6 h to obtain the polyamide acid (PAA)/ nano-Al_2_O_3_ composite. To prepare PI/Al_2_O_3_ nanocomposite films, a certain amount of PAA/Al_2_O_3_ solution is painted on glasses and degassed in vacuum. Finally, the samples are putted into a vacuum oven and the thermal imidization process is: 80 °C - 2 h, 100 °C - 1 h, 140 °C - 1 h, 180 °C - 1 h, 240 °C - 0.5 h, 300 °C - 0.25 h in turn. The synthesized nanocomposite films of the thickness between 35 ~ 50 μm are picked out as the samples of the corona-aging tests while the 175 ~ 350 μm ones are prepared for the space charge distribution tests (PEA tests) and the trap level tests (TSC tests).

### Characterization

The chemical structure of the PI/Al_2_O_3_ nanocomposite films are analyzed by Fourier transform infrared spectroscopy (FT-IR, Thermo Nicolet 5700, USA) between 400 and 4000 cm^−1^. The samples are analyzed at 2 cm^−1^ resolution and 10 scans co-averaged. Before measuring, the background of the atmosphere is measured and subtracted from each spectrum.

Micro morphology of the films are observed by field emission scanning electron microscope (FE-SEM, ZEISS Sigma, Germany). Cross-section of the samples for FE-SEM observation are obtained by brittle fracture in liquid nitrogen. Then the nano-Al_2_O_3_ particles exposed at the cross-section are eroded by soaking the nanocomposite films in diluted hydrochloric acid (0.5 mol/L HCl) for 4 hours and the small pits left on the surface show the size and distribution of the nanoparticles. All the samples are sputtered with gold on the surfaces to avoid charge accumulation during observation.

### TSC tests

After the long term corona-aging process, the corona-aged films were cleaned with anhydrous alcohol and then desiccated in the vacuum oven at 120 °C for 2 hours. Before the TSC tests, the samples are sputtered with gold at two sides of the corona-aged areas. The tested sample is first polarized under 3 kV/mm at 120 °C for 30 min then cooled down to −20 °C in the speed of -10 °C/min. After a delay of 3 min, the sample is depolarized for 10 min to release the polarization charges. Finally it is linearly heated in 4 °C/min with the depolarization current recorded. The thicknesses of the tested 0 wt%, 2 wt% and 5 wt% films are 336 μm, 350 μm and 270 μm for unaged samples, 220 μm, 265 μm and 180 μm for 80 °C long-term corona-aged samples, 210 μm, 200 μm, and 175 μm for 160 °C long-term corona-aged samples.

## Additional Information

**How to cite this article**: Yang, Y. *et al.* “Thermal Stabilization Effect” of Al_2_O_3_ nano-dopants improves the high-temperature dielectric performance of polyimide. *Sci. Rep.* **5**, 16986; doi: 10.1038/srep16986 (2015).

## Supplementary Material

Supplementary Information

## Figures and Tables

**Table 1:** The energy depth obtained by half-width method.

Samples	Trap depth E [ev]		
	Unaged	Corona-aged at 80 °C	Corona-aged at 160 °C
0 wt%	0.5921	0.7885	0.8616
2 wt%	0.5381	0.5465	0.6118
5 wt%	0.5818	0.4997	0.7793
