# Supplementary material for: “Thermal Stabilization Effect” of Al2O3 nano-dopants improves the high-temperature dielectric performance of polyimide
Source: Sci Rep. 2015 Nov 24;5:16986. doi: 10.1038/srep16986 (PMC4657051; doi:10.1038/srep16986)
Supplement: Supplementary Information [file srep16986-s1.doc]

# Supplementary Information

“Thermal Stabilization Effect” of Al_2_O_3_ nano-dopants improves the high-temperature dielectric performance of polyimide

Yang Yang^1^, Jinliang He^1,*^, Guangning Wu^2^, and Jun Hu^1^

^1^State Key Lab of Power Systems, Department of Electrical Engineering, Tsinghua University, Beijing, 100084, China

^2^School of Electrical Engineering, Southwest Jiaotong University, Chengdu 610031, China

Correspondence and requests for materials should be addressed to J.L.H. (email: [hejl@tsinghua.edu.cn](mailto:hejl@tsinghua.edu.cn).)

**Supplementary Note 1: FT-IR** **spectrum**

The FT-IR spectrum curves of 2 wt% and 5 wt% samples are shown in Fig. S1. In order to reveal the detailed characteristic peaks of the curves, only the spectrum from 500 to 2500 cm^-1^ is presented here. The FT-IR spectrum curves of both 2 wt% and 5 wt% samples show the characteristic peaks of aromatic imide including symmetric C=O (1780 cm^-1^), asymmetric C=O (1720 cm^-1^) et al which indicate the imidization reaction on both of the two samples and the formation of aromatic imide. In addition, there are almost no absorption peaks near 1650 cm^-1^, where the characteristic peak of symmetric C=O (the carbonyl group in polyamide acid) is located. It can be concluded that the polymerization and imidization reaction are completed and there are almost no residual polyamide acid (PAA).


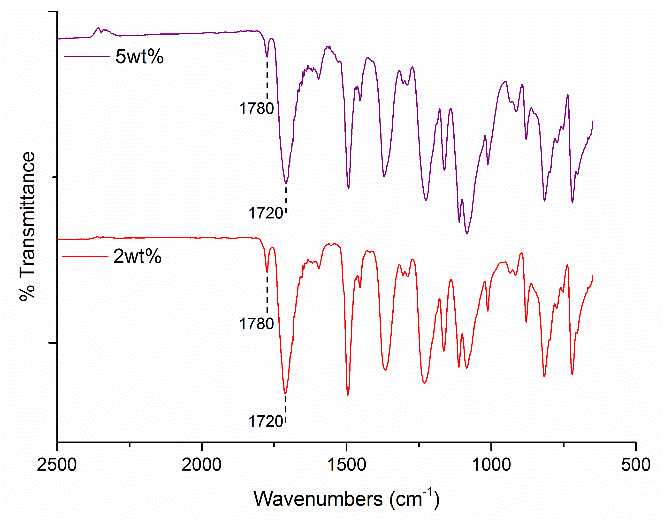


**Supplementary Fig. S1.** **FT-IR spectrum of the PI/Al_2_O_3_ nanocomposite films with 2 wt% and 5 wt% nano-Al_2_O_3_.**

**Supplementary Note 2: Surface and cross section SEM images**

Take the high nano-doping-concentration (5 wt%) samples as examples. The surface SEM image and the cross-section SEM image are shown in Fig. S2 (a) and (b), respectively. The surface image demonstrates the smooth surface morphology of the prepared PI/Al_2_O_3_ nanocomposite films. Few white spots represent the nanoparticles near the surface. The eroded cross-section SEM image of the sample represents the size and distribution of the Al_2_O_3_ nanoparticles. It can be observed that the nanoparticles are few tens of nm in diameter and there is few aggregation of the particles. The prepared PI/Al_2_O_3_ nanocomposites are homogeneously dispersed nanodieletrics.


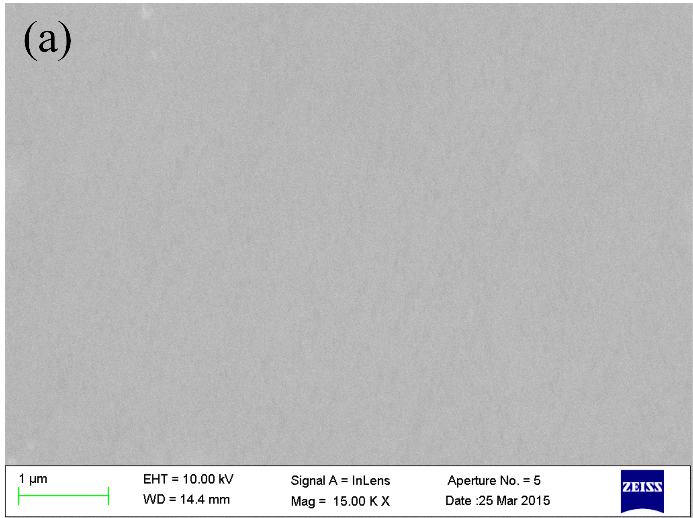


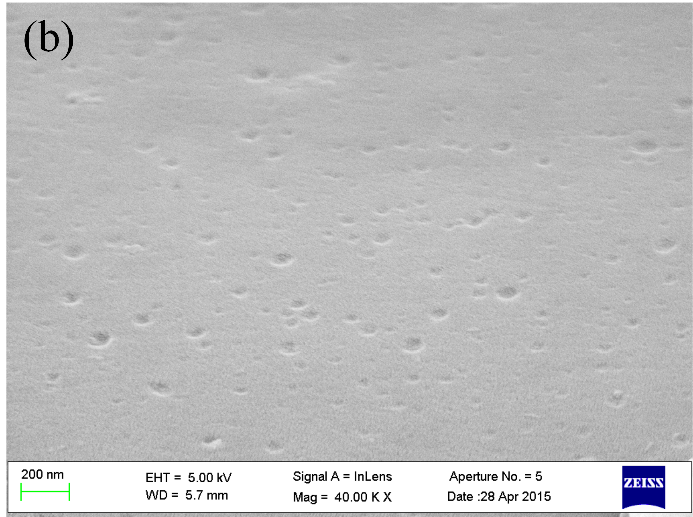


**Supplementary Fig. S2.** **The surface and** **etched cross section SEM images of 5 wt% (take the high dopant concentration films as examples) PI/Al_2_O_3_ nanocomposite films.** (a) surface image, (b) etched cross section image.

**Supplementary Note 3: Corona-aging tests**

In corona-aging tests, sample films (uncoated) are cut into square pieces of 2 cm×2 cm, and the rod-plate electrode is employed according to IEC std. 60343-1991 (shown in Fig. S3 (a)). In order to avoid the influence of air gap between the rod electrode and sample surface, samples with breakdown point inside the contact area (shown in Fig. S3 (b)) are treated as invalid. Experimental equipment is shown in Fig. S4. The rod-plate electrode is put into a thermostatic dryer to keep the temperature at constant. A bipolar high-frequency high-voltage (HV) square wave pulse, which is generated by the high-voltage amplifier (TREK Model 30/20A, slew rate greater than 750 V/μs), is applied on the samples. The square wave input signal is given by the function generator (Tektronix AFG3102C) and the output voltage and current are monitored by the oscilloscope (Tektronix DPO 2024).


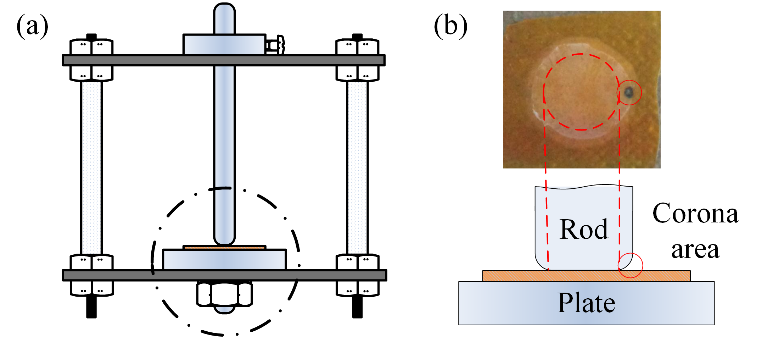


**Supplementary Fig. S3.** (a) IEC std. 60343-1991 rod-plate electrode (6 mm ± 0.3 mm diameter with the sharp edge removed to leave a 1mm radius). (b) Corona discharges and breakdown occur under the chamfering of the rod electrode.


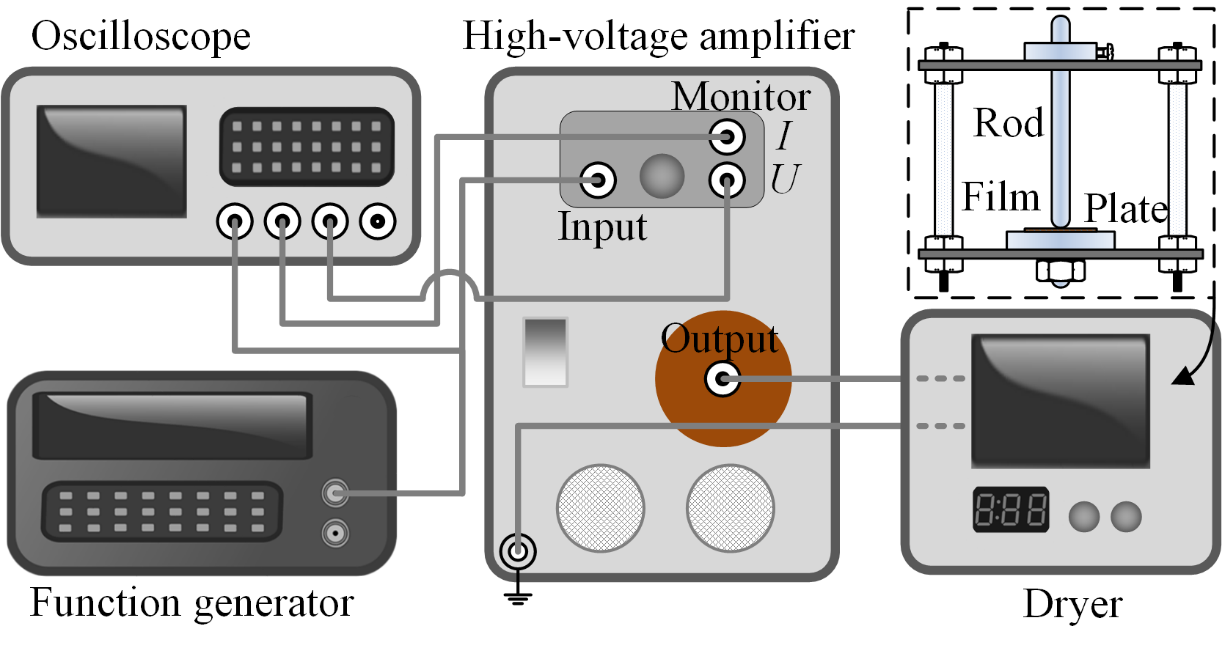


**Supplementary Fig. S4. Experimental equipment.**

**Supplementary Note 4: Derivation of the** ***L*-*E*_av_ inverse power model**

According to the “lifetime (*L*) - voltage (*V*)” inverse power model, which is widely used in corona resistant lifetime evaluation, the “lifetime (*L*) - the average electric field intensity (*E_av_*)” inverse power model was put forward. For the films of the same thickness, there is a power relationship between corona resistant lifetime and the amplitude of applied square wave voltage as shown in Eq. (S1)^1,2^

$L=C_{V}V^{-n_{V}}$ (S1)

where $C_{V}$ and $n_{V}$ are the fitting parameters. Assuming that the thickness of a sample is *d*, then the average electric field intensity *E_av_* can be obtained and Eq. (S1) become

$L=C_{V}d^{-n_{V}}E_{av}^{-n_{V}}$. (S2)

It is widely accepted that the insulation life of a kind of dielectric depends on the electric field intensity with no direct relationships to the thickness nor the applied voltage^1^. For the lifetime of a kind of dielectric:

$C_{V}d^{-n_{V}}=constant$ (S3)

$L=C_{E}E_{av}^{-n_{V}},C_{E}=C_{V}d^{-n_{V}}$ (S4)

Eq. (S4) reveals the relationship between corona resistant life and the average electric field intensity which illustrates the corona resistance of the material nature.

**Supplementary Note 5: Energy level of carrier traps**

The measured thermally stimulated current (TSC) curves are shown in Fig. S5. In order to determine the average trap depths of the samples by half-width method, the required parameters obtained from the current curves are shown in Table S1. The calculated average trap depths of the samples are illustrated in Fig. S5(d). Because of the different thicknesses of the films (details are shown in Method section of the paper) which is unavoidable during the preparation process, there are some numerical differences of the depolarization current between the tested samples.

As is demonstrated by the PEA tests, the space charge in the corona-aged samples is the electronic homo-charge injected from the electrodes. Thus only the electronic charge carriers are considered in the TSC tests and the following analysis of the trap levels. In this case, the cathode electrode is ohmic while the anode electrode is blocking in contact^3^. Considering that the trap levels of polymers should be continuous with the distribution function *N_t_*(*E*), the thermally stimulated depolarization current density can be calculated as^3^:

$J(T)=\frac{el^{2}}{2d}\int_{E_{v}}^{E_{c}} f_{0}(E)N_{t}(E)e_{n}(E,T)\text{e}^{-\frac{1}{\beta}\int_{T_{0}}^{T} e_{n}(E,T)\text{d}T}\text{d}E$ (S5)

where *e* is electronic charge quantity, *f_0_* is the initial occupancy of a trap level and is a constant, *E* is the trap depth. $e_{n}(E,T)=v\exp(-E_{t}/kT)$ is the emission rate of electrons at trap level *E* and temperature *T*. *k* is the Boltzmann constant, *d* is the sample thickness and *β* is the heating rate. *v* is commonly called the frequency factor or attempt-to-escape frequency, typically 10^12^ s^-1^. *E_v_* and *E_c_* are the valence band top energy and conduction band bottom energy, respectively. In order to obtain *N_t_* from the TSC spectra directly, a new function is introduced^4^:

$G1(E,T)=v\text{e}^{-E/kT}\text{e}^{-\frac{1}{\beta}\int_{T_{0}}^{T} v\text{e}^{-E/kT}\text{d}T}$ (S6)

which determines the weighted contribution of an electron at trap level *E* to the current at temperature *T*. From Equation (S5) and (S6), Ref. 3 provides a new method based on the modified isothermal discharge current theory^4^ and MATLAB numerical calculation to directly obtain the trap level distribution from TSC measurement. The MATLAB program provided in Ref. 3 is introduced here to determine the trap level density [(eV·m^3^)^-1^] curves.

Because of the continuous distribution of the polymer dielectrics, the distribution probability of the traps of different energy level couldn’t be obtained. Considering that the trap level density *N_t_*(*E*) [(eV·m^3^)^-1^] obtained above is the differential of trap density to energy level, the trap density *n_t_*(*E*) below a certain energy level *E* can be determined by integration of the trap level density *N_t_*(*E*) from the valence band top energy *E_v_* to *E*:

$n_{t}=\int_{E_{v}}^{E} N_{t}(E)\text{d}E$. (S7)

It is easy to understand that the trap density *n_t_*(*E*) is a monotonically increasing function of *E*. As the energy level *E* increase from *E_v_* to *E_c_*, the integration of Equation (S7) will include all the traps and the trap density *n_t_*(*E*) increases to the total number (density) of the traps. However the total density of the all the traps may differ from each other for different samples and it is meaningful to calculate the distribution probability of trap density by dividing the trap density *n_t_*(*E*) with the total density *n_t_*(*E_c_*) especially regarding the comparison between different kinds of samples. The calculated distribution probability curves of trap density are shown in Fig. S6.

From Fig. S6, the proportion of the traps below a certain energy level *E* can be obtained. Consistent with the average trap depth calculated by half-width method, the distribution probability of trap density of the unaged samples show little difference among the three nano-dopants concentrations which is due to the low-concentration layer on the nanocomposite films surface. Regarding the samples long-term corona-aged at 80 °C, the 0wt% curve is below the other two curves at low energy level (<1.0 eV) and beyond the others at high energy level (>1.0 eV). By comparing the corona-aged and unaged owt% samples in Fig. S6(a) and (b), it is obvious that the proportion of deep (high energy level) traps increases after corona-aging. These significant differences indicate the shallow traps of nanocomposite films introduced by nanoparticles and the higher degradation degree of the pure PI nanocomposites. The 5wt% sample shows the highest proportion of trap density at low energy level below 0.9 eV and shows lower proportion than the 2wt% one. Higher nano-dopants concentration introduces more shallow traps which further demonstrates that the shallow traps are caused by the nanoparticles. For the samples corona-aged at 160 °C, a significant shift of the curve to the higher energy level can be observed with regard to the 5 wt% samples. The 5 wt% nanocomposite films are seriously degraded at 160 °C while the 2 wt% ones keep the trap depth distributing at low energy level. These results agree well with the proposed mechanisms of the “thermal stabilization effect” that the phase interface regions with shallow traps will overlap with each other in the case of high nano-dopants concentration and high temperature conditions. Then the low-resistance channels with long mean free path^5^ for electrons would be formed and the electronic carriers will get accelerated and attack the molecular chains to result in defects which act as deep traps in the dielectrics^6,7^.


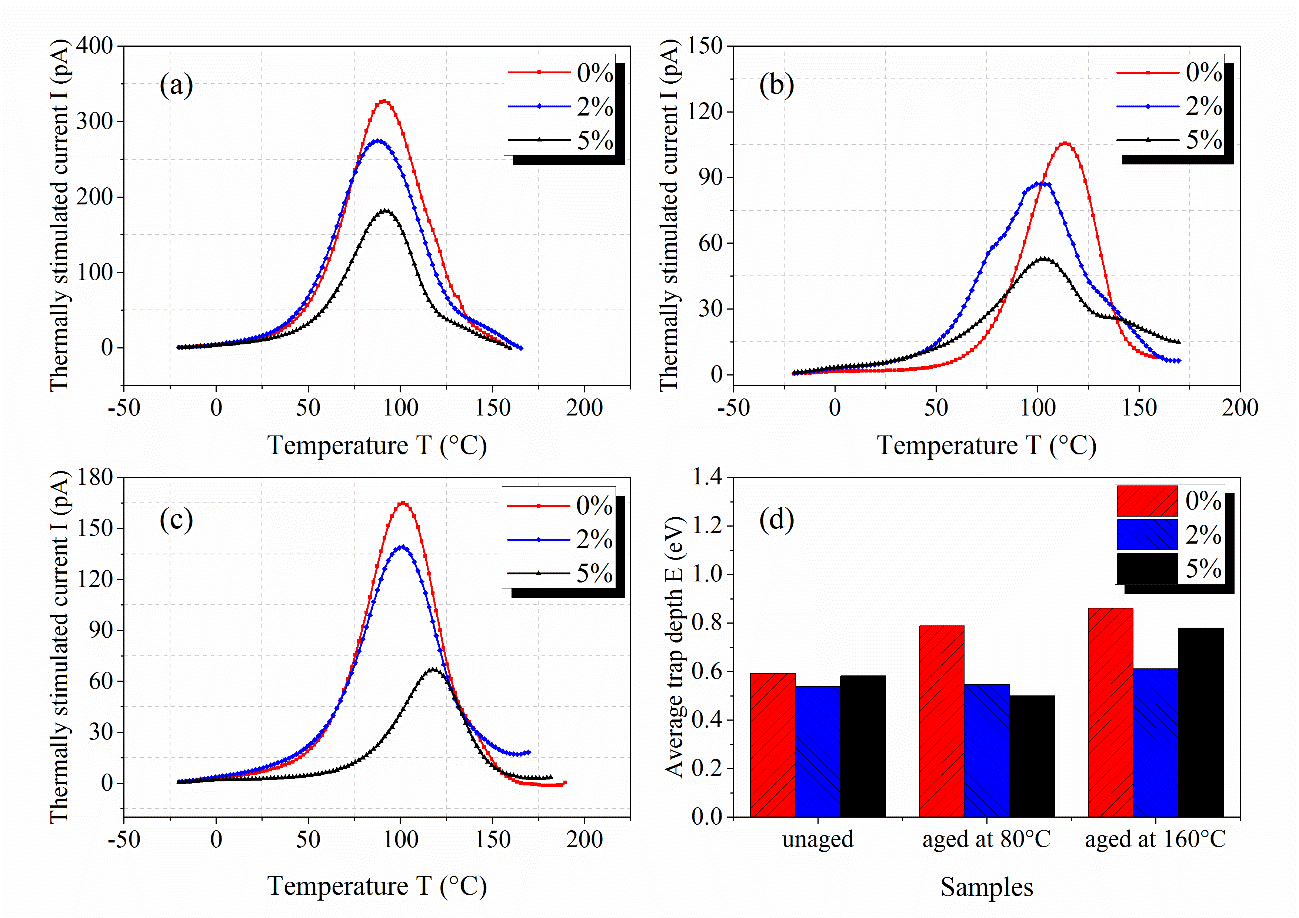


**Supplementary Fig. S5.** **TSC curves of PI/Al_2_O_3_ nanocomposite films with different nano doping concentrations and temperature conditions of long term corona-aging.** (a) Unaged samples. (b) Samples corona-aged at 80 °C. (c) Samples corona-aged at 160 °C. (d) The trap depth of samples calculated the half-width method.

**Supplementary Table. S1. The required parameters required by half-width method.**

| Samples | Unaged | | | Aged at 80 °C | | | Aged at 160 °C | | |
| --- | --- | --- | --- | --- | --- | --- | --- | --- | --- |
|  | *T*^a^*_m_*/°C | *T*^b^*_L_*/°C | *T*^c^*_H_*/°C | *T_m_*/°C | *T_L_*/°C | *T_H_*/°C | *T_m_*/°C | *T_L_*/°C | *T_H_*/°C |
| 0wt% | 91.58 | 67.96 | 115.78 | 113.70 | 91.30 | 131.70 | 102.33 | 77.67 | 112.50 |
| 2wt% | 87.90 | 62.09 | 113.65 | 101.43 | 70.04 | 124.69 | 100.44 | 74.86 | 123.41 |
| 5wt% | 91.15 | 65.38 | 113.93 | 103.31 | 73.83 | 134.20 | 116.70 | 95.32 | 136.83 |

^a^The temperature corresponding to the peak value of TSC curve.

^b^The lower temperature at half peak of the curve.

^c^The higher temperature at half peak of the curve.


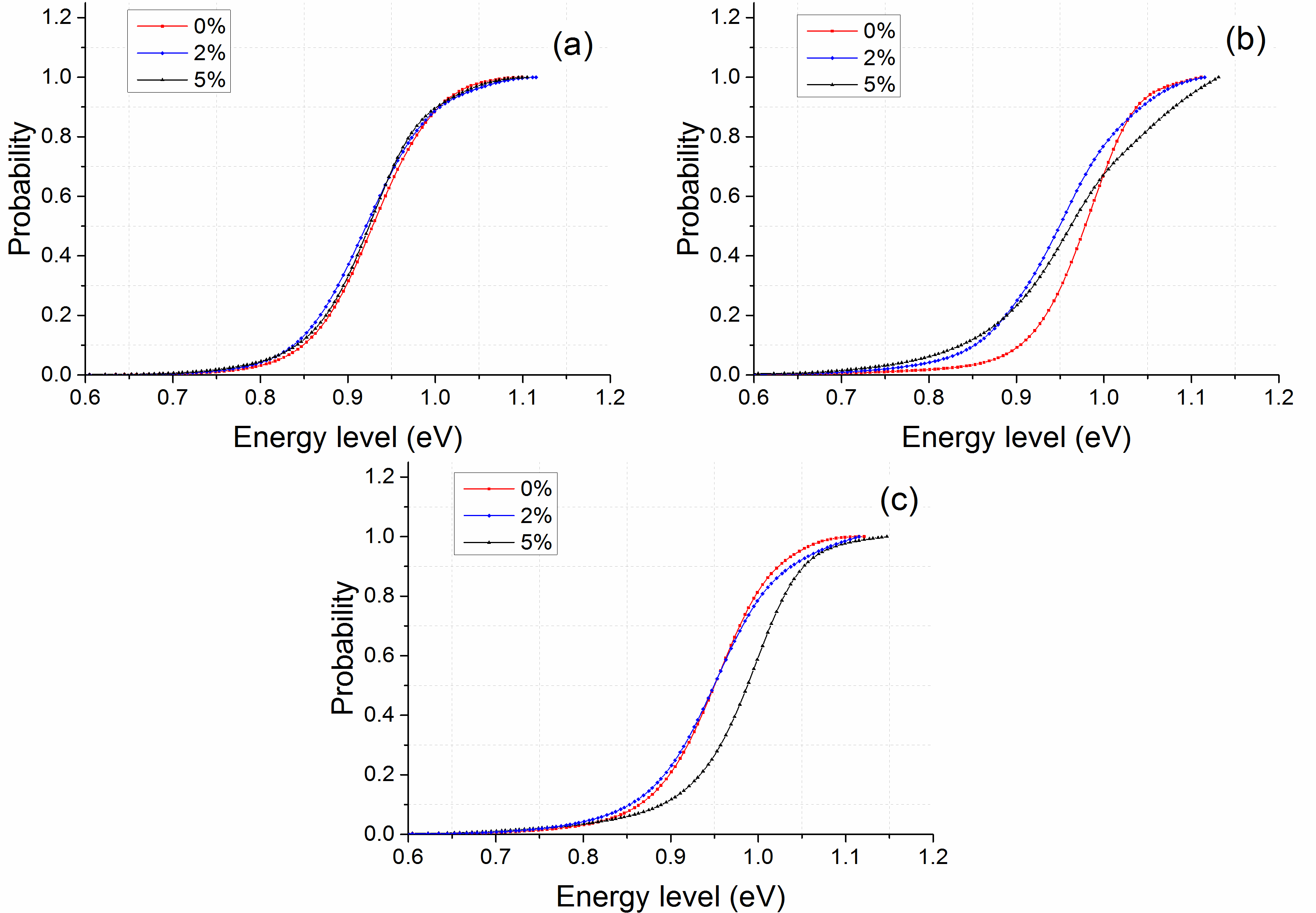


**Supplementary Fig. S6. Calculated trap density distribution probability curves of PI/Al_2_O_3_ nanocomposite films with different nano doping concentrations and temperature conditions of long term corona-aging.** (a) Unaged samples. (b) Samples corona-aged at 80 °C. (c) Samples corona-aged at 160 °C.

**Supplementary References**

1. Cacciari, M. & Montanari, G C. Electrical life threshold models for solid insulating materials subjected to electrical and multiple stresses. II. Probabilistic approach to generalized life models. *Ieee T. Dielect. El. In.* **27,** 987-999 (1992).
2. Kaufhold, M., Borner, G., Eberhardt, M. & Speck, J. Failure mechanism of the interturn insulation of low voltage electric machines fed by pulse-controlled inverters. *Ieee Electr. Insul. M*. **12,** 9-16 (1996).
3. Tian, F. et al. Theory of modified thermally stimulated current and direct determination of trap level distribution. *J. Electrostat*. **69,** 7-10 (2011).
4. Lei, Q., Tian, F., Yang, C., He, L. & Wang, Y. Modified isothermal discharge current theory and its application in the determination of trap level distribution in polyimide Films. *J. Electrostat*. **68,** 243-248 (2010).
5. Wu, K., Dissado, L. A. & Okamoto, T. Percolation model for electrical breakdown in insulating polymers. *Appl. Phys. Lett*. **85,** 4454-4456 (2004).
6. Kao, K. C. New Theory of electrical discharge and breakdown in low-mobility condensed insulators. *J. Appl. Phys*. **55,** 752-755 (1984).
7. Meunier, M., Quirke, N. & Aslanides, A. Molecular modeling of electron traps in polymer insulators: chemical defects and impurities. *J. Chem. Phys*. **115,** 2876-2881 (2001).
